# NVVC/NHJ Durrer prizes 2015

**DOI:** 10.1007/s12471-016-0827-5

**Published:** 2016-04-04

**Authors:** E. E. van der Wall

**Affiliations:** Netherlands Society of Cardiology/Holland Heart House, Moreelsepark 1, 3511 EP Utrecht, the Netherlands

**Keywords:** Durrer prizes, Netherlands Society of Cardiology (NVVC), Annual Congress, Netherlands Heart Journal

## Abstract

At the annual 2016 Spring Congress of the NVVC, the Durrer prizes were awarded to the authors of two of the best original articles published in the year 2015, one paper being more basically oriented and one paper being more clinically oriented. This annual tradition has existed since the year 2006.

For the 10th time in a row, the Netherlands Heart Journal (NHJ), the official journal of the Netherlands Society of Cardiology (NVVC), awarded the Durrer Prizes to two outstanding NHJ articles published in the year 2015. In 2006, it was thought appropriate by the NVVC Board to set up a special publication prize in order to stimulate the submission of outstanding scientific articles to NHJ. Two articles per year are selected, one article with a more basically oriented character and one with a main clinical focus [1–5]. Apart from originality and scientific quality, one of the criteria for selection is the number of citations in international journals.

The NHJ publication prize carries the name of one of the fathers of Dutch Cardiology, Professor Dirk Durrer (1918–1984), Head of the Department of Cardiology in the ‘Wilhelmina Gasthuis’, Amsterdam, who performed pioneering work in the field of electrical activation of the heart in the 1960s and 1970s [6, 7]. Furthermore, Dirk Durrer founded the Interuniversity Cardiology Institute of the Netherlands (ICIN) in 1972, of which he was director until 1983. In 2008, the Durrer Center for Cardiovascular Genetics was founded with the final aim to produce outstanding international research [8].

From a total of 149 articles published in NHJ in 2016, we selected as best basically oriented article: *The revised role of TGF-β in aortic aneurysms in Marfan syndrome. *Authors: Franken R, Radonic T, den Hartog AW, Groenink M, Pals G, van Eijk M, Lutter R, Mulder BJ, Zwinderman AH, de Waard V; on behalf of the COMPARE study group. Neth Heart J. 2015;23:116–21. doi: 10.1007/s12471-014-0622-0 [9]. This study showed in 99 patients with Marfan syndrome that patients responding to losartan therapy with a reduction of the plasma TGF-β level had higher baseline TGF-β levels and a higher aortic root dilatation rate. Most likely, TGF-β levels may be considered to be a readout of the disease state of the aorta. The authors propose that increased angiotensin II is the initiator of aorta dilatation and is responsible for increased TGF-β levels in Marfan syndrome. This article has been cited, among others, in the Canadian Journal of Cardiology, the International Journal of Cardiology, and Circulation-Cardiovascular Genetics.

As best clinical article we selected: *High survival rate of 43 % in out-of-hospital cardiac arrest patients in an optimised chain of survival. *Boyce LW, Vliet Vlieland TP, Bosch J, Wolterbeek R, Volker G, van Exel HJ, Heringhaus C, Schalij MJ, Goossens PH. Neth Heart J. 2015;23:20–5. doi: 10.1007/s12471-014-0617-x [10]. This study showed in 242 patients with out-of-hospital cardiac arrest (OHCA) that a survival rate of 43 % after OHCA is achievable after one year of hospital discharge. Witnessed cardiac arrest, cardiac cause of arrest, initial cardiac rhythm and Glasgow Coma Scale ≥ 13 were associated with higher survival. This study has been cited, among others, in PLOS One, the International Journal of Cardiology, Resuscitation, and the Scandinavian Journal of Trauma Resuscitation & Emergency Medicine.

At the annual spring meeting of the NVVC, held at the Congress Centre ‘De Leeuwenhorst’ in Noordwijkerhout on 31 March 31 and 1 April 2016, the first authors of these two articles received an educational grant provided by the NVVC. We would like to congratulate the authors on their awards and thank them for sending their excellent work to NHJ. With the Durrer Prizes, we again hope to stimulate young investigators to send their best papers to the NHJ.
